# Cytotoxicity Induction by the Oxidative Reactivity of Nanoparticles Revealed by a Combinatorial GNP Library with Diverse Redox Properties

**DOI:** 10.3390/molecules26123630

**Published:** 2021-06-14

**Authors:** Shenqing Wang, Xiliang Yan, Gaoxing Su, Bing Yan

**Affiliations:** 1 School of Chemistry and Chemical Engineering, Shandong University, Jinan 250100, China; wang_shen_qing@163.com; 2 Key Laboratory for Water Quality and Conservation of the Pearl River Delta, Institute of Environmental Research at Greater Bay, Ministry of Education, Guangzhou University, Guangzhou 510006, China; yanxiliang1991@gzhu.edu.cn; 3 School of Pharmacy, Nantong University, Nantong 226001, China; sugaoxing@ntu.edu.cn

**Keywords:** nano-combinatorial chemistry, gold nanoparticle, redox, nanotoxicity, QSAR

## Abstract

It is crucial to establish relationship between nanoparticle structures (or properties) and nanotoxicity. Previous investigations have shown that a nanoparticle’s size, shape, surface and core materials all impact its toxicity. However, the relationship between the redox property of nanoparticles and their toxicity has not been established when all other nanoparticle properties are identical. Here, by synthesizing an 80-membered combinatorial gold nanoparticle (GNP) library with diverse redox properties, we systematically explored this causal relationship. The compelling results revealed that the oxidative reactivity of GNPs, rather than their other physicochemical properties, directly caused cytotoxicity via induction of cellular oxidative stress. Our results show that the redox diversity of nanoparticles is regulated by GNPs modified with redox reactive ligands.

## 1. Introduction

The rapid increase in the application of nanomaterials in consumer products, biomedical materials, and industries, is a result of the vast diversity of their structural properties. Gold nanoparticles (GNPs) have been the object of investigations for usages as contrast agents for computed tomography, as drug delivery vehicles and as agents for photothermal cancer therapy, despite having no FDA approval yet. This presents a major challenge for effective and timely screening for their potential adverse effects in biological and environmental systems [1,2,3,4]. The inherent properties of nanoparticles, including size, shape, chemical composition, surface functionalization, solubility, hydrophobicity, surface charge and agglomeration state may all affect their interactions with biomolecules and cells [5,6,7]. Therefore, these physicochemical factors can determine the bioactivity and toxicity of nanoparticles [8,9,10,11]. 

Previous reports have focused on the influences of certain physicochemical properties of nanoparticles, such as size, surface chemistry and shape or core composition, on cytotoxicity [12,13,14]. More solid conclusions were achieved for the effects of nanoparticle size and surface chemistry. The effects of core composition on cellular perturbations were recently reported by us [15], and studies on the influence of shape have also been published [16,17,18]. However, as an intrinsic property of all nanoparticles, the effects of the redox reactivity of nanoparticles on cellular perturbations have not been systematically studied. Attribution of physicochemical determinants of nanoparticles to their toxicity is complex and requires systematical investigations because all these factors may participate in biological regulation simultaneously [19,20,21]. Therefore, it is difficult to solve this problem without systematic and isolated studies because of the lack of comparability. Facing this challenge, we developed a nano-combinatorial nanoparticle library approach and solved issues related to surface chemistry, size and core material. These systematic studies provided more convincing results [22,23,24].

In this study, we explored the relationship between the redox reactivity of nanoparticles and cellular perturbations using a combinatorial GNP library with diverse redox properties. In this systematic work, the redox-diversified library was first synthesized by incorporating a ferrocene moiety and organic ligand molecules with diversified structures. Cellular studies then demonstrated that the redox reactivity of GNPs, rather than other physicochemical properties, causes cytotoxicity via induction of cellular oxidative stress.

## 2. Results and Discussion

### 2.1. Combinatorial GNP Library with Diversified Redox Properties

**Scheme 1:**
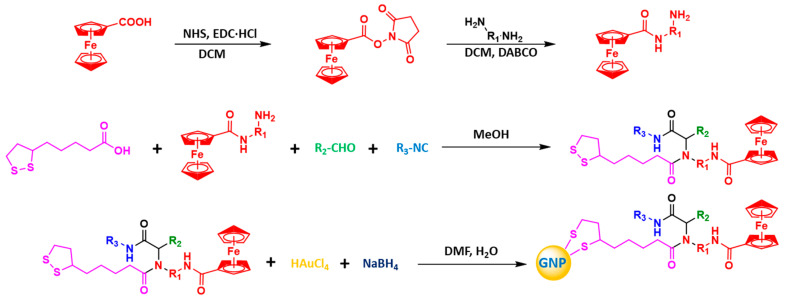
Synthesis of the combinatorial GNP library. Using the Ugi multicomponent reaction, ligand molecules with a ferrocene group and various molecules structures were synthesized before a combinatorial GNP library with various redox activities was constructed using these ligands.

In order to model the naturally diverse redox properties of nanoparticles, we designed a combinatorial GNP library. A ferrocene group was introduced into each ligand molecule on the surface of GNPs. Ferrocene can undergo redox reactions repeatedly with oxidative or reductive species in human cells [25,26,27]. These reactions are through the reversible transformation of Fe(II) and Fe(III) with the loss and gain of electrons. Moreover, the redox activity of the ferrocene group is probably affected by the ligand structure so that redox activities of GNPs may be modulated. In line with these designs, we initiated the synthesis of a combinatorial GNP library following Scheme 1.

**Figure 1:**
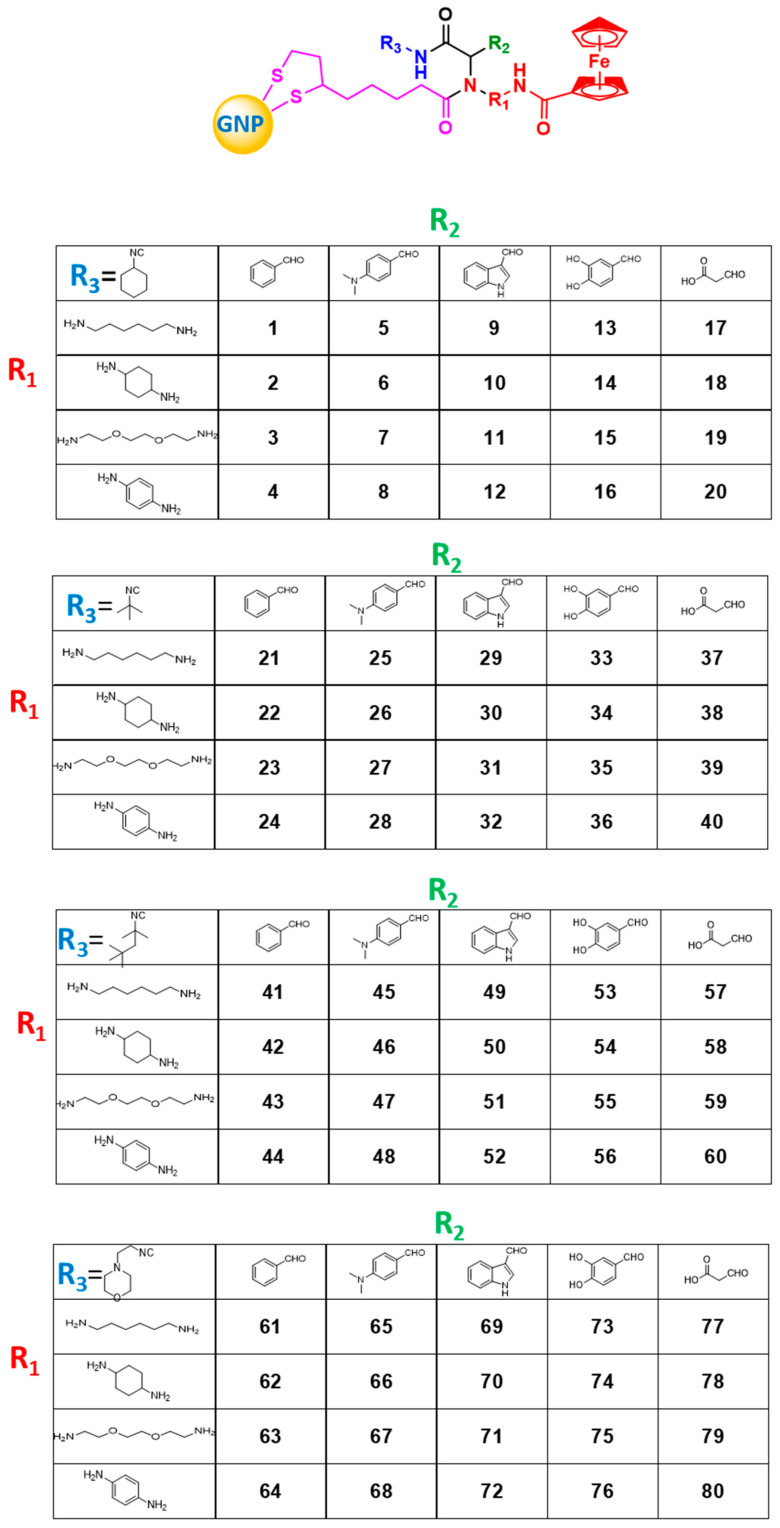
The numbering of 80 GNPs in the combinatorial GNP library. Based on the strategy of combinatorial chemistry, the surface physicochemical properties of GNPs were regulated by diverse ligand molecules. Five aldehyde compounds, four amino compounds and four isonitriles were used in the synthesis, resulting in a total of 80 GNPs.

The key to success in this study was to first design diverse ligand molecules that could modulate the ferrocene group and therefore the redox activity of the GNPs. With this in mind, we introduced linear and cyclic substituent structures to diversify the influence of molecule geometry and introduced aromatic ring structures with different substituents to reflect the influence of π electron density. The introduction of heteroatoms reflected the effect of hydrophilicity and hydrogen bond acceptors, the amino and carboxyl groups reflected the influence of charge and the hydroxyl groups reflected the influence of hydrogen bond donors (Figure 1). 

### 2.2. Characterization of Physiochemical Properties of the GNP Library

**Figure 2:**
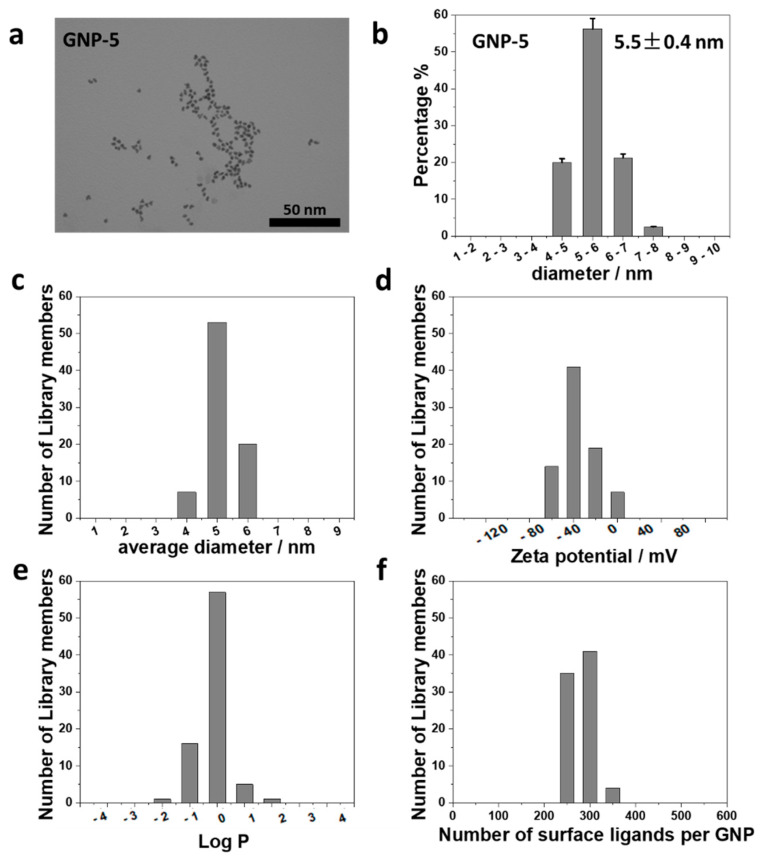
An overview of the basic physical and chemical properties of the GNP library. (**a**) TEM characterization of library gold nanoparticles (**GNP-5** as an example) with a magnification of ×100,000. (**b**) The average particle size distribution for **GNP-5** was 5.5 ± 0.4 nm. (**c**) The average particle size distribution for all GNPs in the whole library. TEM data for GNPs in the whole library were in the range of 4.5~6.2 nm. (**d**) The distribution of zeta potential values of the GNP library. (**e**) The distribution of log *p* values for the whole GNP library determined by the “shaking flask” method. (**f**) The distribution of surface ligand numbers per GNP across the whole GNP library determined by ICP-MS.

The GNP library was fully characterized for the GNPs’ physicochemical properties. Morphologies of GNPs were determined by TEM (Figure 2a and Figure S1). The GNPs had a spherical shape and uniform diameter. The average diameter of the entire library was 5.6 ± 0.5 nm (Figure 2b). In solution, GNPs were well dispersed with a slight aggregation tendency. Electrostatic and electrodynamic properties of GNPs were assessed by their zeta potential, which was on average −24.8 ± 9.1 mV (Figure 2d and Figure S2b). 

The hydrophobicity of a GNP determines its ability to penetrate cell membranes or bind to important biomolecules. This reactivity is characterized by GNPs’ water/octanol participation coefficient or log *p* value. The log *p* values of all GNPs were determined with a modified “shaking flask” method [28,29,30]. Log *p* values of the 80 GNPs in this library were between −1.0 and 1.0 and the average was 0.59 ± 0.24 (Figure 2e and Figure S2a).

### 2.3. Direct In Situ Quantification of the GNP Surface Modifications

**Scheme 2:**
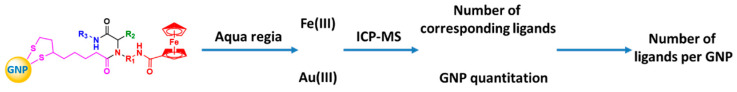
One step determination of the number of ligands per GNP. The concentrations of Au(III) and Fe(III) were quantitatively determined by ICP-MS. The concentration of total GNPs was determined according to the Au content, and the total number of ligands was determined according to the Fe content. The loaded ligands per nanoparticle could then be obtained.

GNP properties, in large part, are determined by the surface ligand molecules or surface chemistry. However, determination of the number of surface ligands is not an easy task, especially in high-throughput screening. In most previous papers, this was not reported. In others, various analytical methods were applied, such as elemental analysis [31], thermogravimetric analyses [32] or cleavage and analysis methods [33]. For a large nanoparticle library or ligand molecules with multiple common elements, the applications of the above methods are limited. In this work, we established a direct, quick and accurate method to obtain both the number of ligands on each GNP and the number of GNPs in solution. By incorporating the ferrocene group in every ligand, we achieved both of the above goals using ICP-MS analysis (Scheme 2).

In our method, the concentrations of Au and Fe were simultaneously determined by ICP-MS in one step analysis, and the number of GNPs and number of ligands were calculated accordingly. The number of loaded ligands per GNP was the ratio of the ligand number to GNP number. For the GNP library, the number of ligands per GNP was in the range of 240~359 (Figure 2f and Figure S2C). Compared with the previous methods, our method involves fewer samples, simpler operation, fewer steps, reduces the possibility of error and is suitable for high-throughput characterization of a large number of nanoparticle samples.

### 2.4. Diverse Redox Activities of the GNP Library and Underlying Mechanisms

**Figure 3:**
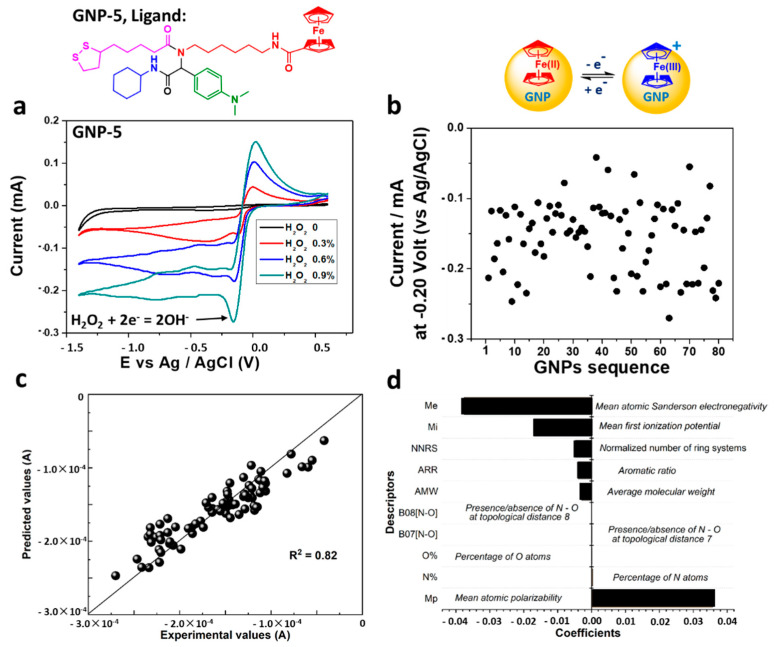
Diverse redox reactivities of the GNP library. (**a**) With **GNP-5** as an example, the reduction activities of GNPs were determined by cyclic voltammetry and electro-catalytic decomposition of hydrogen peroxide (H_2_O_2_ + 2e^−^ = 2OH^−^). Nanoparticles with the same concentration (1.0 mg/mL, 10 μL) were added to the surface of the glassy carbon electrode. The catalytic activity of GNPs was determined by the decomposition rate (current density) of hydrogen peroxide under specific voltage (**GNP-5** as a model). (**b**) Diverse redox reactivities of the GNPs in the library. Absolute values of the experimental data for the whole library were in range of 0.04~0.27 mA. (**c**) Correlations between experimental reduction values and multi-linear regression model predictions. The line of y = x and the coefficient of determination (R^2^) are also shown in the plot. (**d**) Contributions of the top 10 Dragon descriptors from the modeling results. The descriptors were ranked based on the coefficients of the model. A description of each descriptor is also shown.

The ferrocene group offers GNPs a unique electron exchange ability. These GNPs can thus be expected to have electrochemical reactivities. Diverse molecule structures of ligands create different microenvironments, so the electrochemical reactivities of GNPs may be highly diverse. Such reactivities of GNPs were determined by cyclic voltammetry using the electrocatalytic reduction of H_2_O_2_. Different GNPs exhibited different intensities of reduction peak currents produced by H_2_O_2_ because of their different H_2_O_2_ reducing abilities. The ferrocene group surrounded by each individual molecular environment determined the redox activity of GNPs (Figure S3). The peak current is defined as the current density at −0.20 V (Ag/AgCl) as shown in Figure 3a. Cyclic voltammetry and the peak currents of all GNPs were experimentally determined (Figure 3b and Figure S4). The peak current values of the GNP library varied, ranging from −0.04 to −0.23 mA, suggesting their different H_2_O_2_ reducing abilities [34,35,36,37]. After uptake by cells, GNPs with a stronger reducing ability will convert more H_2_O_2_ into OH^-^, which is relatively nontoxic [38,39,40,41]. In this process, the levels of ROS in cells are inhibited better, thus avoiding the occurrence of oxidative stress and cell damage.

Furthermore, we explored how the structural features of surface ligands actually regulated the reduction activity of GNPs by analyzing the resulting predictive model. Based on the coefficients of each variable in the model, the top seven ranked descriptors were selected and are shown in Figure 3d. The high ranking of a descriptor use indicated its critical contribution to the final modeling and the reduction activity of GNPs. Therefore, the reduction activity of GNPs was mostly relevant to the mean atomic electronegativity and mean atomic polarizability. Electronegativity is the tendency of an atom to attract shared electrons to itself, and polarizability is defined as the dipole moment induced in the atom in response to the application of an electric field. Obviously, the distribution of electrons or electron clouds inside atoms greatly affects the reduction activity of GNPs, while these properties are significantly enhanced by ligand structural diversity. The properties (physical and chemical properties and reactivity) of molecules are determined by their molecular orbitals and the arrangement of electrons in the molecular orbitals. The structural diversity of ligands inevitably affects the composition of molecular orbitals and the energy levels of the HOMO and LUMO, modulating the physical and chemical properties and reactivity of molecules and GNPs. Previous studies have also shown that the electronegativity and polarizability of atoms are closely related to the reduction activity of materials [42,43]. The model constructed here and the corresponding mechanism analysis can help us design nanomaterials with the required reduction activity in the future. Based on the theoretical calculation of the quantum chemistry, the weight of many influencing factors can be summarized and analyzed so as to realize the effect prediction of nanomaterials and the auxiliary design of new nanomaterials.

### 2.5. Redox Activities of GNPs Induce Cytotoxicity via Regulating the Cellular Oxidative Stress 

**Figure 4:**
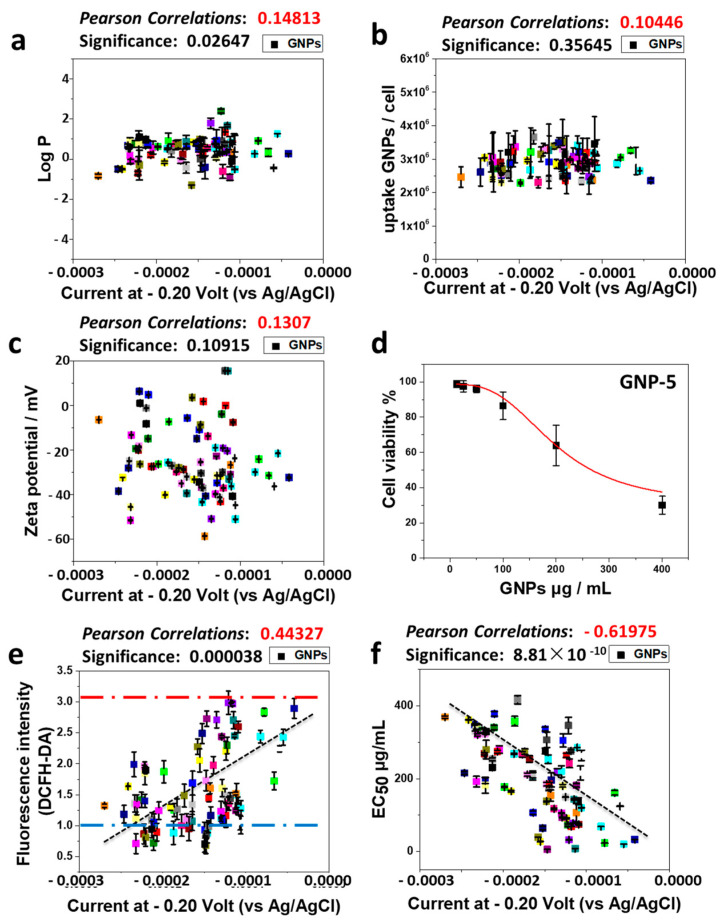
The relationship between the redox activity of GNPs and their physicochemical or biological properties. (**a**) The change of redox activity of GNPs did not alter the hydrophobicity of GNPs. (**b**) Furthermore, there was no effect on the cell uptake of GNPs. (**c**) The redox activity was independent of the surface charge state (zeta potential) of GNPs. (**d**) With **GNP-5** as an example, the dose-dependent cytotoxicity of A549 cells is shown. (**e**) There was a positive correlation between the level of cellular oxidative stress and the oxidative activity of GNPs. Blue dotted line: negative control; red dotted line: positive control; black dotted line: change trend. (**f**) A negative correlation between EC50 values (or a positive correlation between cytotoxicity) and the oxidative activity of GNPs. Black dotted line: change trend.

In order to examine whether the redox activity and other physicochemical properties of GNPs play a role in inducing cellular perturbations, we determined cellular uptake (Figure 4b and Figure S2d), cellular oxidative stress (Figure S2e) and cytotoxicity along with their relationships with GNP properties. The GNP-induced changes in relative fluorescence intensity of the DCFH-DA probe reflected the level of cellular oxidative stress. There was a clear trend that cellular oxidative stress was positively correlated with the oxidative properties of GNPs (Figure 4e). GNP-induced cytotoxicity was determined with the CellTiter-Glo^®^ assay (Figure 4d and Figure S5). GNP concentration that induced 50% cell death (EC50) was used to reflect cytotoxicity. The redox activity of the GNP library was correlated with cytotoxicity, with more oxidative GNPs inducing more cytotoxicity. It is well-known that H_2_O_2_ is one of the important ROS species in cells [44,45,46]. Combining the above results, GNP-induced cellular oxidative stress was positively correlated with cytotoxicity (the lower the EC50 values, the higher the cytotoxicity). Excessive ROS in cells may result in random oxidation of nucleic acids, proteins and lipids, which may lead to the loss of their normal functions by changing the structure of these biomolecules [47,48,49,50,51]. When this kind of damage exceeds the self-healing ability of cells, it induces programmed cell death.

To examine whether other basic physicochemical properties of GNPs play a role in GNP-induced oxidative stress in cells, the effects of hydrophobic properties, zeta potential, cellular uptake and surface ligand loading were analyzed. Our results showed that the number of surface ligands (Figure S6), hydrophobic properties (Figure 4a and Figure S7), zeta potential (Figure 4c and Figure S7) and cellular uptake (Figure 4b) did not have any correlation with the induction of cellular oxidative stress or cytotoxicity in A549 cells. Therefore, the diverse redox activities of GNPs were the only reason for cytotoxicity via induction of cellular oxidative stress.

The 80 GNP library members were all modified by organic ligand molecules. Although ligands were diverse, they hardly altered some of the basic physical properties, such as the hydrophobicity and the zeta potential. However, these structural diversities were enough to change the redox reactivities of the GNPs and, therefore, the induction of cellular oxidative stress and cytotoxicity. The GNP library approach has been used previously to develop cancer cell-targeting nanoparticles [22] and cellular oxidative stress-enhancing nanoparticles [52].

## 3. Materials and Methods

### 3.1. Chemicals

Lipoic acid (97%), petroleum ether (99%), triethylamine (98%), tetrahydrofuran (97%), ethanol (99%), ethyl acetate (99%), dichloromethane (97%), 1-octanol (≥85%), N-dimethylformamide (≥97%), sodium bicarbonate (≥95%), sodium chloride (90%), sodium hydroxide (≥97%), hydrochloric acid (98%), sodium borohydride (95%), hydrogen peroxide (30%) and chloroauric acid (99%) were purchased from Sigma-Aldrich (St. Louis, MO, USA).

### 3.2. Synthesis of Ligands

In an ice bath, 11.5 g (0.05 mol) of ferrocenecarboxylic acid was mixed with 100 mL of dichloromethane (DCM). Under vigorous stirring, 7.0 g (0.06 mol) of N-hydroxysuccinimide (NHS) and 11.5 g (0.06 mol) of 3-ethylcarbodiimide hydrochloride (EDC·HCl) were added to the reaction system. The reaction was stirred for 4–6 h in an ice bath and monitored by TLC. After reaction, filtration was carried out and a DCM solution of (**1**) was obtained.

In an ice bath, 8.5 g (0.075 mol) of triethylenediamine was added to 50 mL of DCM. Then, 24.6 mL of 1,4-cyclohexanediamine was added and stirred until the reaction system was uniformly stabilized. The DCM solution (**1**) was added dropwise to the reaction system within 2 to 3 h. The reaction was maintained continuously for 24 h. The reaction system gradually became viscous and the reaction was monitored by TLC. After reaction, the solvent was removed by rotary evaporation, and the crude product was washed repeatedly with water and purified by column chromatography. An orange-red solid product (**2**) was obtained.

Intermediate (**2**) (0.75 g, 5 mmol), benzaldehyde (0.50 mL, 5 mmol), cyclohexyl isocyanide (0.60 mL, 5 mmol) and lipoic acid (1.03 g, 5 mmol) were added to 10 mL of methanol and reacted at 320 K for 48 h. The reaction was monitored by TLC. After reaction, the solvent was removed by rotary evaporation, the crude product was purified by column chromatography and ligand 1 was obtained. 

**Ligand 1** was an orange-red solid with a yield of 54.11%; ^1^H NMR (500 MHz, DMSO-d_6_) δ (ppm) = 8.45 (s, 1H), 8.05 (s, 2H), 7.9 to 7.5 (m, 5H), 4.53 (m, 1H), 3.34 (m, 1H), 3.01 (t, 1H), 2.6 (m, 3H), 2.1 to 1.1 (m, 27H); C_27_H_41_N_3_O_3_S_2_; ESI-MS: m/z 730.3 (M+1)^+^.

### 3.3. Synthesis of the GNP Library 

We mixed an orange solution containing 0.0032 mmol of ligand (**Ligand 1**) in 20 mL of DMF, 12.5 mg of HAuCl_4_·3H_2_O, 0.6 mg of ammonium chloride and 0.95 mg of sodium citrate in 2.25 mL of water at room temperature. Then, in an ice bath with vigorous stirring, 15 mg of sodium borohydride in 15 mL of water was slowly added to the reaction system through peristaltic pumping over 60 min. The solution color became dark and then red gradually. GNPs were named from **GNP-1** to **GNP-80** according to the types of modified ligands. After the Ugi reaction, 80 different ligands were obtained. Each ligand molecule had only one ferrocene group (one iron atom) with the “labeling” of the ligands undertaken by using the Fe atom.

### 3.4. Measurements of Zeta Potentials of GNPs

GNPs were suspended in aqueous solution with sonication. The zeta potentials of GNPs were measured at room temperature using a Malvern Zetasizer instrument (Nano ZS90, Malvern Instruments Ltd., Worcestershire, UK). All samples were measured at the same concentration. Each sample was measured in triplicate. The samples were diluted with Milli-Q water with a pH value of 6.83 when measuring zeta potential. Amine groups in the starting materials were converted to amides after the reactions so that there were no amine groups in the final products.

### 3.5. Determination of Loaded Ligands on GNPs 

GNPs were suspended in aqueous solution with sonication. Then, 50 μL of GNP solution was mixed with 1.0 mL of aqua regia. The sample was completely digested and diluted with Milli-Q. The contents of gold and iron were determined by ICP-MS (Varian 820-MS). The GNP concentration was determined by the Au concentration, and the ligand concentration was determined by the Fe concentration.

GNPs can be approximately regarded as spheres. The gold atom number in a spherical GNP with a specific particle size can be calculated (**m_Au_ ·*N*_A_**= ***M*_Au_**, **m_Au_ · n_Au_ = m_GNP_ = *ρ*_Au_ ·V_GNP_**). Therefore, the results of ICP-MS can reflect the number of GNPs in a specific volume of GNP solution. In addition, the number of iron atoms is equal to the number of ligands (**n_Fe_ = n_Ligand_**).

Number of ligands = (*C*_Fe_/*M*_Fe_) (*C*_Au_ *M*_Au_)^−1^ · (*ρ*_Au_ ·V_GNP_ *N*_A_ *M*_Au_) (1)

An example of such analysis can be shown for **GNP-5:** the volume of **GNP-5** was 87 nm^3^ (**V** = 4/3 **· π · r^3^**) since the average particle diameter was 5.5 nm (Figure 2a). *ρ***_Au_** = 19.32 kg/m^3^. Based on the ICP-MS results, the Au content was 146.51 ppb and the Fe content was 2.73 ppb (all results after deducting the background value). Using Equation (1), we were able to determine that the number of ligands loaded on **GNP-5** was 337.

### 3.6. TEM Images Characterization

TEM images of the GNPs were taken using a JEOL-1011 transmission electron microscope (JEM, Tokyo, Japan) at 100 KV. The images were acquired using an AMT 2k CCD camera. Image data were analyzed by ImageJ.

### 3.7. Hydrophobicity Measurements of GNPs

An equal volume of deionized water and n-octanol were mixed and stirred for 24 h. The mixture solution was allowed to stand quietly. After the separation, the upper layer was the water-saturated n-octanol, and the lower layer was the n-octanol-saturated aqueous solution. The two phases were collected and used in the next step. 

Hydrophobicity of GNPs was measured according a previously reported method. Firstly, 0.3 mg of GNPs was dispersed with 1.0 mL of n-octanol-saturated aqueous solution. Secondly, 1.0 mL of water-saturated n-octanol was added. Thirdly, the mixtures were vigorously shaken on a shaker for 24 h. After that, the mixtures were left to stand quietly until the two phases were completely separated. Each phase was carefully collected and dried at 120 °C under vacuum for 4 h. Finally, 500 μL of fresh aqua regia was added to digest GNPs over 12 h. After diluting, gold concentrations in each phase were measured by ICP-MS. Log *p* values of GNPs were calculated according to the gold concentrations in the two phases.

### 3.8. Preparation of GNP Solution for Cell-Based Experiments

The aqueous dispersion of the gold nanoparticles was sonicated for 15 min. Sterile water was then added to dilute GNPs to a concentration of 1.0 mg/mL, and uniformly dispersed stock solutions were obtained. After sterilization by batch sterilization, stock solutions were stored at 4 °C before use. For cell experiments, after sonication, stock solutions were diluted with cell culture medium to desired concentrations and vortexed before adding to plate wells.

### 3.9. Cytotoxicity Test

A549 cells were cultured with RPMI 1640 medium and, respectively, supplemented with 10% fetal bovine serum, 100 U/mL penicillin and 100 µg/mL streptomycin. Then, 100 μL of A549 cells in the logarithmic growth phase at a concentration of 6 × 10^4^ cells/mL was seeded to each well of 96-well plates. After incubation for 24 h, the medium was removed, and fresh, complete medium containing different concentrations of GNPs (i.e., 12.5 μg/mL, 25 μg/mL, 50 μg/mL, 100 μg/mL, 200 μg/mL, and 400 μg/mL) was added to each well. The cells were then incubated with GNPs for 48 h. After removing the medium and washing with PBS twice, 50 μL of the complete medium and 50 μL of the CellTiter-Glo^®^ assay working fluid were added in sequence. After gently shaking for 2 min, the plates were incubated for 10 min at room temperature in the dark. Then, 70 μL of the mixture solutions in each well were transferred to another set of white, opaque 96-well plates, and the fluorescence signal intensity was detected by a plate reader (VICTORTM X2, Perkin Elmer, Waltham, MA, USA). Deducting the background signals, cell viability was calculated. Each experiment was done in parallel three times.

### 3.10. Cellular Oxidative Stress Test

A549 cells were seeded in 24-well plates (6 × 10^4^ cells/well). After 24 h incubation, the medium in each well was removed. GNPs in cell culture medium (500 μL, 50 μg/mL) were added to each well and incubated with cells for 24 h. The cells were then washed with PBS twice and 2,7-dichlorodihydro-fluoresceindiacetate (DCFH-DA, 10 μM) in medium were added. After incubation for 30 min in the darkness, cells were washed with PBS three times, treated with trypsin, and collected for measurements with a fluorescence microplate reader. Groups with only cell culture medium added and H_2_O_2_ (500 nM) were set as negative and positive controls, respectively. Cellular oxidative stress was determined using the DCFH-DA method. In the absence of GNPs, cellular oxidative stress values with and without H_2_O_2_ were used as positive and negative controls.

### 3.11. Cell Uptake Experiment 

A549 cells in the logarithmic growth phase were seeded in 24-well plates with a density of 4 ×10^5^ cells/well. After 24 h incubation at 37 °C, each well was washed twice with 500 μL of pre-warmed, serum-free RPMI1640 medium. Then, 500 μL of RPMI1640 complete medium containing 50 μg/mL GNPs was added to each well.

After another 24 h incubation at 37 °C, each well was washed three times with cold PBS and 200 μL of trypsin was added to each well. After 4 min digestion, 200 μL of the RPMI1640 complete medium was added to stop this process. Cells in each well were counted and 200 μL of the cells was collected. Fresh aqua regia was then added to completely digest the GNPs overnight at 37 °C. After diluting, gold contents in each well were measured using ICP-MS. Each experiment was done in parallel three times.

### 3.12. Determination of Redox Reactivity of GNPs

The hydrogenation ability of GNPs was evaluated by electrocatalysis of H_2_O_2_ to OH^−^ using an electrochemical workstation (CHI660c, CH Instruments, Inc.). A glassy carbon electrode was used as the working electrode, the Pt electrode as the counter electrode and the Ag/AgCl electrode as the reference electrode. Each GNP from the library with the same concentration (1.0 mg/mL, 10 μL) was added to the polished glassy carbon electrodes. The electrochemical reactions were performed in a 0.01 M PBS solution with 0.3%, 0.6% and 0.9% H_2_O_2_ at a scan rate of 20 mV·s^−1^. The initial potential, the maximum potential and the minimum potential were set at 0 V, 0.6 V and −1.4 V, respectively.

### 3.13. Correlation Analysis

Based on the definition of the *Pearson correlation coefficient*, the correlation between different characteristics (physical and chemical properties, biological effects) of GNPs (quotient of covariance and standard deviation between different characteristics) was calculated, and the correlation degree between these characteristics was analyzed.

$$\rho_{X,\;Y}=\frac{cov\left(X,\;\;Y\right)}{\sigma X\sigma Y}$$

### 3.14. Structure Activity Relationship

To explore the quantitative relationships between the nanostructures and the redox properties of GNPs, we used the structural information (i.e., Dragon descriptors) of the surface ligands to develop a quantitative structure–activity relationship (QSAR) model to predicting their redox reactivities. Here, we used a multiple linear regression method, called the least absolute shrinkage and selection operator (LASSO) model, to build the QSAR model. As a classic machine learning method, LASSO can enhance the prediction accuracy and interpretability of the resulting models through performing both variable selection and regularization. In the present LASSO model, the regularization parameter was set to 0.0001 and the maximum number of iterations set to 100,000. As a result, a total of 58 descriptors were selected to fit the model. Both model construction and prediction were performed by the machine learning library in Python, scikit-learn v0.19.2. The correlation between experimental and predicted reduction activity values is shown in Figure 3c. The high determination coefficient (R2 = 0.82) showed that the machine learning model correctly predicted GNPs’ redox activities.

## 4. Conclusions

In this study, we explored the induction of cytotoxicity by a combinatorial GNP library with diverse redox activity. In this systematic work, we established that the cytotoxicity was directly caused by the oxidative reactivity, rather than other properties, of GNPs. There are two important implications of this study. First, a simple electrochemical characterization of nanoparticles should in the future be used to predict the basic toxicity risk of nanoparticles. Second, screening of a GNP library yields an array of nanoprobes, which can be embedded into human cells to probe cellular mechanisms or generate cell models with various oxidative stresses. Both of these findings will accelerate nanomedicine and nanotoxicity studies significantly. Through regulation of redox activity, GNPs may regulate cells to the appropriate level of oxidative stress to enforce a synergistic effect, which may further enhance the applications of GNPs in more biomedical fields, such as imaging, drug delivery, diagnosis and clinical treatment.

## Data Availability

Supplemental data are provided in the Supplementary Materials, Figures S1–S6.

## Supplementary Materials

The following are available online. Figure S1: TEM images of 80 GNPs in the whole library. Figure S2: The characterization of physicochemical properties and biological effects of 80 GNPs in the whole library. Figure S3: When the ligand does not contain ferrocene substituents, GNPs do not show obvious redox activity. Figure S4: The redox activities of 80 GNPs in the whole library were characterized by cyclic voltammetry. Figure S5: The dose response curves of 80 GNPs in the whole library. Figure S6: The relationship between the redox activity of 80 GNPs and their hydrodynamic diameter or number of ligand uploads. Figure S7: The relationship between the cellular effects of 80 GNPs and hydrophobic property or surface charge.

## Sample Availability

Samples of the compounds and nanoparticles are available from the authors.

## Abbreviations

DMF	N,N-Dimethylformamide
GNP	Gold nanoparticle
ICP-MS	Inductively coupled plasma massspectrometry
PBS	Phosphate Buffered Saline
SHE	Standard hydrogen electrode
TEM	Transmission Electron Microscope
TLC	Thin Layer Chromatography
