# Supplementary material for: Cytotoxicity Induction by the Oxidative Reactivity of Nanoparticles Revealed by a Combinatorial GNP Library with Diverse Redox Properties
Source: Molecules. 2021 Jun 14;26(12):3630. doi: 10.3390/molecules26123630 (PMC8231810; doi:10.3390/molecules26123630)
Supplement: Supplementary file 1 [file molecules-26-03630-s001.zip › molecules-1231866-supplementary.pdf]

Supporting Information

Cytotoxicity induction by the oxidative reactivity of nanoparticles revealed by a combinatorial GNP library with diverse redox properties

Shenqing Wang, Xiliang Yan, Gaoxing Su, Bing Yan*

Contents

Figure S1. TEM images of 80 GNPs in the whole library.

Figure S2. The characterization of physicochemical properties and biological effects of 80 GNPs in the whole library.

Figure S3. When the ligand does not contain ferrocene substituents, GNPs do not show obvious redox activity.

Figure S4. The redox activities of 80 GNPs in the whole library were characterized by cyclic voltammetry.

Figure S5. The dose response curves of 80 GNPs in the whole library.

Figure S6. The relationship between the redox activity of 80 GNPs and their hydrodynamic diameter or number of ligand uploads.

Figure S7. The relationship between the cellular effects of 80 GNPs and hydrophobic property or surface charge.

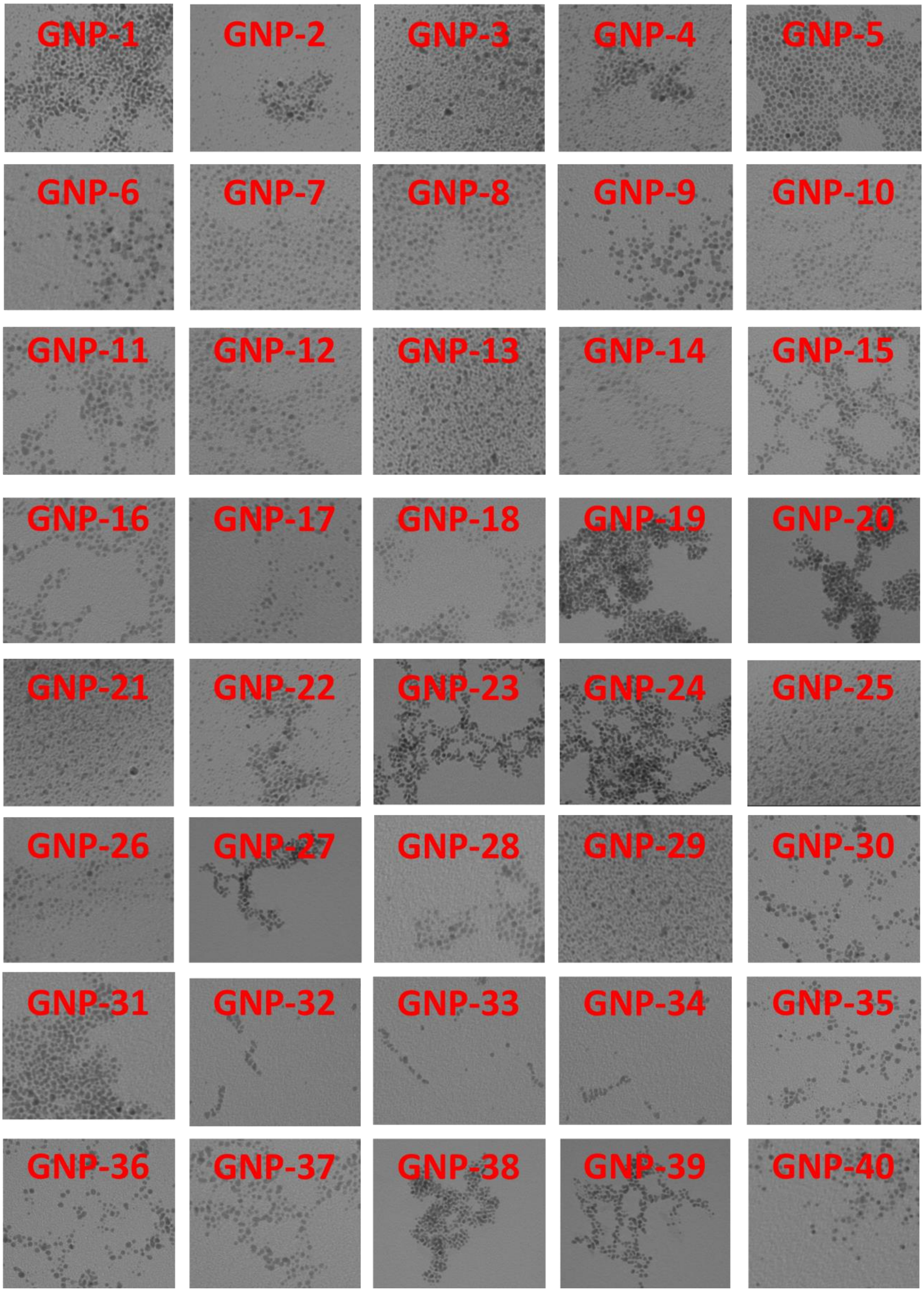
GNP-1
GNP-2
GNP-3
GNP-4
GNP-5
GNP-6
GNP-7
GNP-8
GNP-9
GNP-10
GNP-11
GNP-12
GNP-13
GNP-14
GNP-15
GNP-16
GNP-17
GNP-18
GNP-19
GNP-20
GNP-21
GNP-22
GNP-23
GNP-24
GNP-25
GNP-26
GNP-27
GNP-28
GNP-29
GNP-30
GNP-31
GNP-32
GNP-33
GNP-34
GNP-35
GNP-36
GNP-37
GNP-38
GNP-39
GNP-40

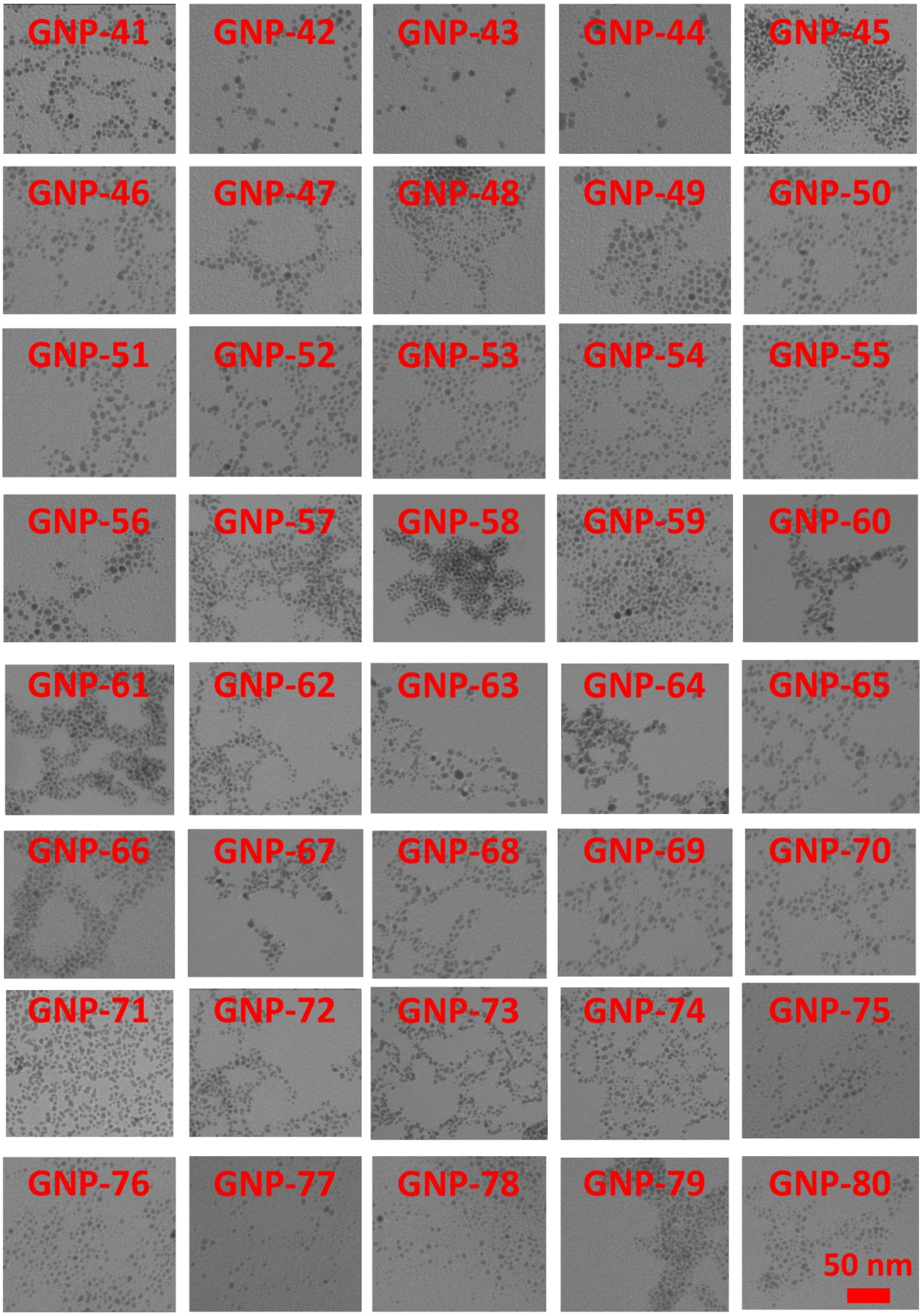

Figure S1. TEM images of 80 GNPs in the whole library.

a

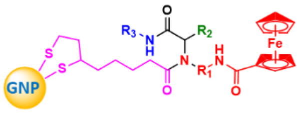

Log P

R3=	R2				
R1	0.99	0.52	-0.5	0.87	0.69
	1.35	0.57	-0.9	0.68	0.77
	0.91	0.3	-0.7	0.59	-0.43
	0.63	-1.3	2.39	1.79	0.42

R3=	R2				
R1	0.07	-0.11	0.02	-0.41	0.47
	0.84	0.45	0.75	0.89	0.27
	0.33	0.91	0.85	0.24	0.37
	0.91	0.51	0.98	0.77	0.32

R3=	R2				
R1	-0.61	0.88	0.74	-0.51	0.78
	-0.44	0.55	0.21	0.18	0.76
	0.58	-0.08	0.33	-0.16	0.77
	0.68	0.76	0.74	0.74	0.76

R3=	R2				
R1	1.66	0.55	0.76	1.09	0.26
	0.82	0.68	1.27	0.23	-0.11
	-0.83	0.52	-0.1	0.62	-0.5
	0.36	0.92	0	0.92	-0.3

b

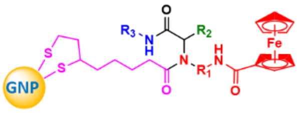

Zeta potential (mV)

R3=	R2				
R1	-8.3	-25.6	-38.5	-39.4	-29.7
	0	-27.3	-30.9	-28.1	-44.7
	-7.3	-31.5	-20.1	-58.6	-32
	-5.6	3.5	-3.9	-50.9	-27

R3=	R2				
R1	-31.4	-40.7	-43.3	-40.6	-33
	-36.3	-43.1	-39.7	-36.7	-32.3
	-25.4	-24.1	-33.2	-28.7	-26.7
	-30	-10.9	-8.7	-14.9	-19.1

R3=	R2				
R1	-37	-45.5	-34.3	-51	-14.9
	-36.3	-19	-27.4	-51.5	-22.7
	-41.2	-35	-31.4	-40.1	-7.6
	-1.2	15.5	4.8	-28	-19.4

R3=	R2				
R1	15.4	-13.8	-18.9	1	-29.9
	6.3	-23.7	-21.5	1.8	-13.3
	-6.4	-24.9	-18.3	-26.3	-32.3
	-20.4	-37	-30.4	-34.7	-26.3

c

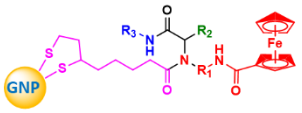

Number of ligands per particle (GNPs)

R3=	R2				
R1	314	337	318	342	309
	295	254	308	332	300
	303	346	263	265	313
	272	297	321	299	330

R3=	R2				
R1	278	334	324	284	284
	317	314	300	276	270
	283	341	312	286	296
	279	259	255	281	333

R3=	R2				
R1	315	341	323	323	335
	274	300	282	359	308
	294	329	352	341	272
	284	342	295	350	240

R3=	R2				
R1	308	328	285	329	307
	324	300	271	277	304
	313	314	293	314	350
	313	257	265	301	277

d

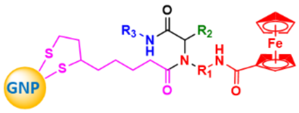

Cellular uptake (GNPs per cell, 50 μg/mL)

R_3= (R$_1$ \ R$_2$)					
	3203090	2672640	2612760	3266390	2305580
	3196570	3366820	2874870	2893540	3339240
	3209580	3097130	2903400	2825340	2423460
	2911730	3385610	2910750	3086770	3661370

R_3= (R$_1$ \ R$_2$)					
	3008870	3452440	2679430	2502230	2439630
	3177830	2812710	3043370	2899370	2358360
	3303980	3048270	2857260	3297360	2372340
	2913930	2878650	3524670	2858500	2893950

R_3= (R$_1$ \ R$_2$)					
	3004190	2425280	2787770	2732160	3493690
	2945470	3413820	3231350	3049000	2726210
	2341410	3281750	3257150	2958440	2965750
	2540950	2758440	2888200	3145130	2712850

R_3= (R$_1$ \ R$_2$)					
	2358900	2983900	2303680	2679890	2861170
	2430210	3044840	2646550	2349300	2912110
	2462020	2878440	2567980	2288390	3038080
	2474080	3148060	2481450	3208090	2708580

e

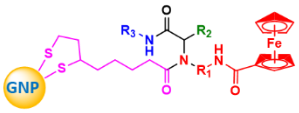

Cellular Oxidative stress (GNPs, 50 μg/mL, relative to normal)

R_3= (R$_1$ \ R$_2$)					
	0.941	0.886	1.186	1.075	1.004
	2.982	1.242	1.242	1.990	1.054
	1.251	2.208	0.870	1.607	1.339
	1.688	2.276	2.297	2.711	1.268

R_3= (R$_1$ \ R$_2$)					
	1.608	1.321	0.887	1.165	2.452
	1.492	1.166	1.234	2.729	2.895
	1.729	2.835	2.055	0.953	1.520
	1.362	0.931	0.679	0.722	2.986

R_3= (R$_1$ \ R$_2$)					
	1.359	1.115	0.702	1.279	2.490
	2.243	1.124	0.895	0.710	2.437
	1.489	1.108	1.724	1.298	2.598
	1.091	1.443	1.054	1.486	1.417

R_3= (R$_1$ \ R$_2$)					
	2.702	1.979	1.847	1.898	2.440
	1.401	1.051	2.428	1.447	1.338
	1.324	1.359	1.958	1.873	1.638
	2.969	0.986	0.855	1.100	0.812

Figure S2. The characterization of physicochemical properties and biological effects of 80 GNPs in the whole library. (a) Log P value, (b) Zeta potential, (c) Number of ligands per GNP, (d) Cellular oxidative stress, (e) Cellular uptake.

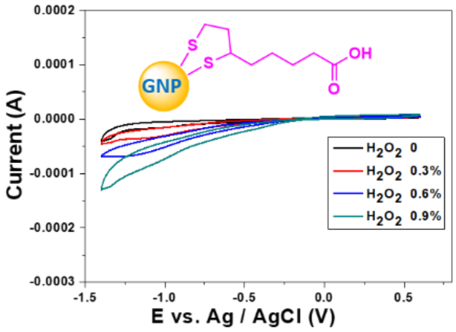

Figure S3. When the ligand does not contain ferrocene substituents, GNPs do not show obvious redox activity. For GNPs, the existence of ferrocene group is a necessary factor to obtain redox activity.

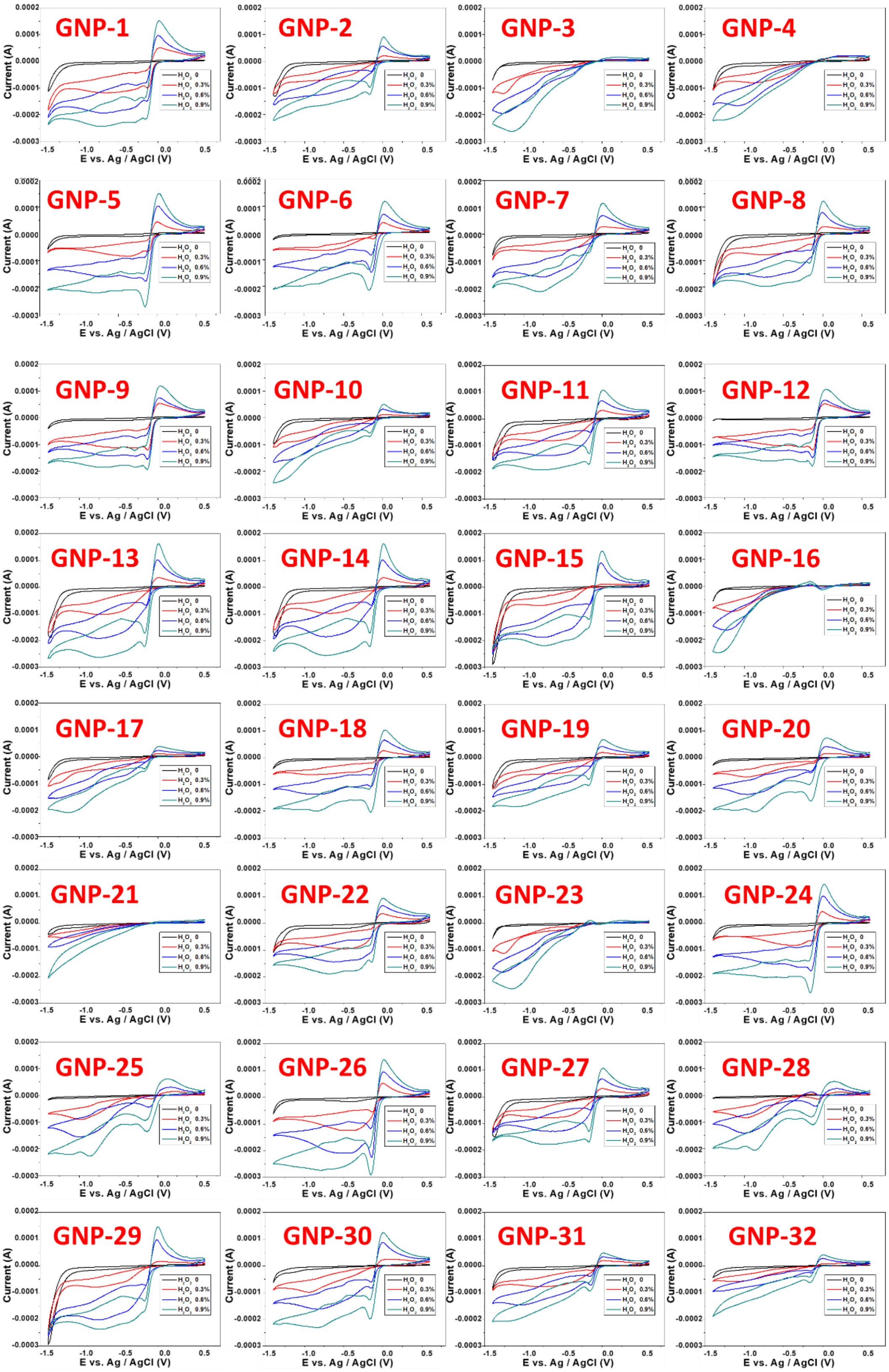
GNP-1
GNP-2
GNP-3
GNP-4
GNP-5
GNP-6
GNP-7
GNP-8
GNP-9
GNP-10
GNP-11
GNP-12
GNP-13
GNP-14
GNP-15
GNP-16
GNP-17
GNP-18
GNP-19
GNP-20
GNP-21
GNP-22
GNP-23
GNP-24
GNP-25
GNP-26
GNP-27
GNP-28
GNP-29
GNP-30
GNP-31
GNP-32
Current (A)
E vs. Ag / AgCl (V)
H_2O_2 0
H_2O_2 0.3%
H_2O_2 0.6%
H_2O_2 0.9%

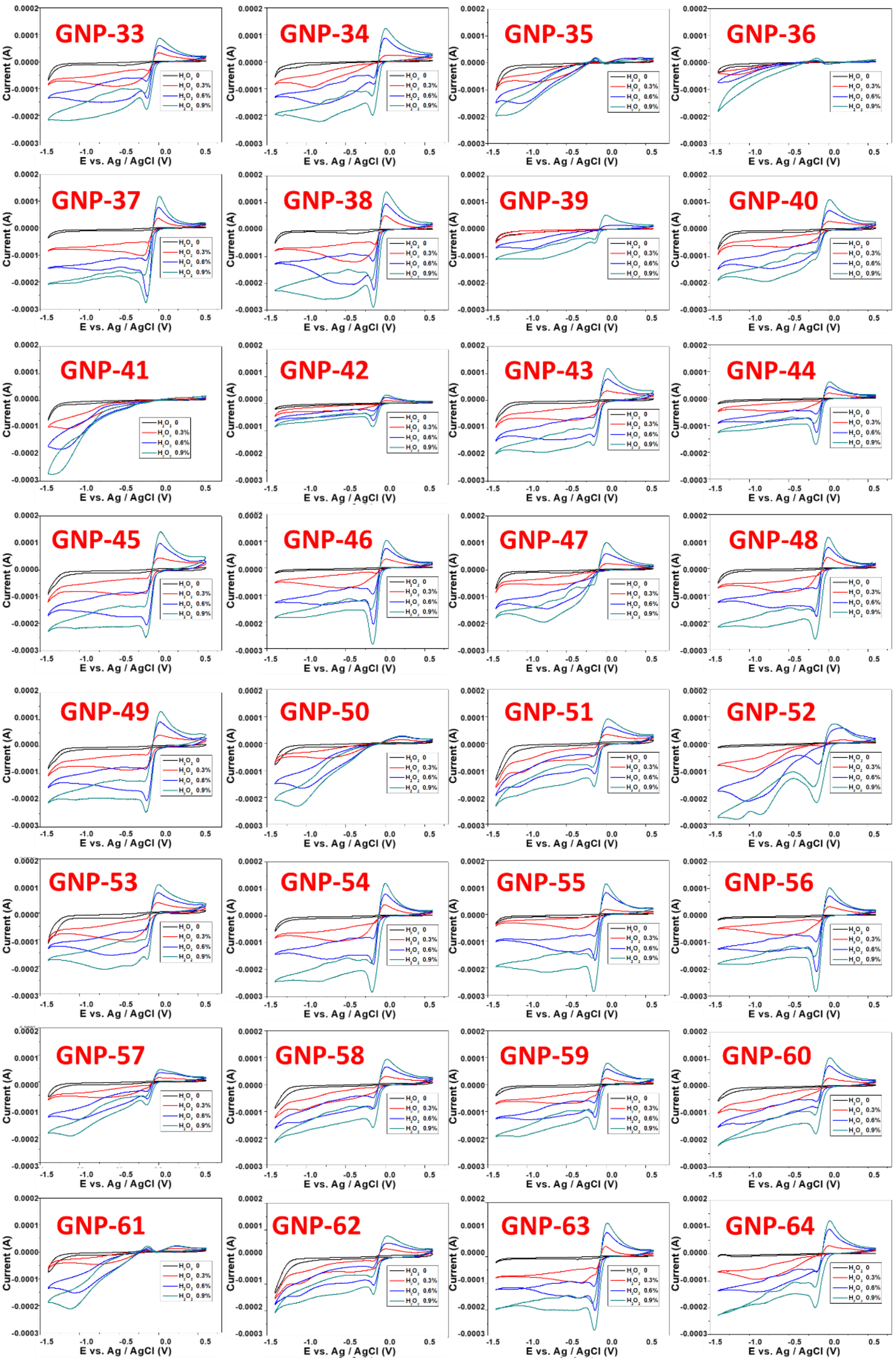
GNP-33
GNP-34
GNP-35
GNP-36
GNP-37
GNP-38
GNP-39
GNP-40
GNP-41
GNP-42
GNP-43
GNP-44
GNP-45
GNP-46
GNP-47
GNP-48
GNP-49
GNP-50
GNP-51
GNP-52
GNP-53
GNP-54
GNP-55
GNP-56
GNP-57
GNP-58
GNP-59
GNP-60
GNP-61
GNP-62
GNP-63
GNP-64
Current (A)
E vs. Ag / AgCl (V)
H_2O_2 0
H_2O_2 0.3%
H_2O_2 0.6%
H_2O_2 0.9%

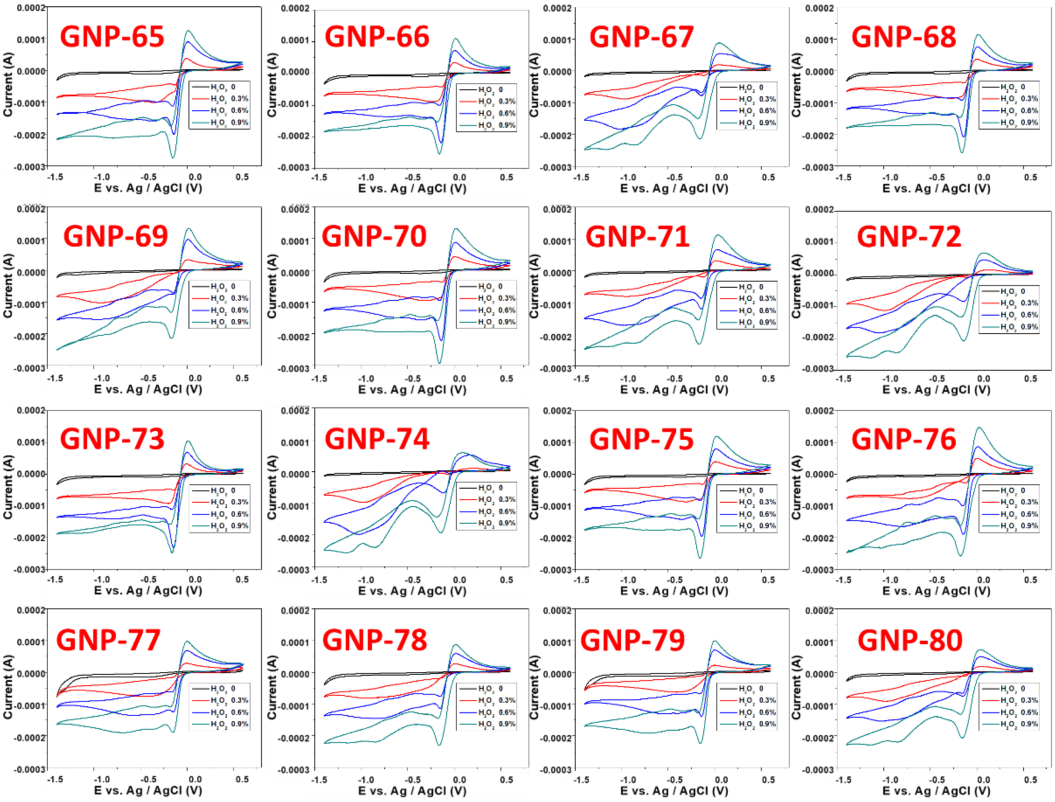

Figure S4. The redox activities of 80 GNPs in the whole library were characterized by cyclic voltammetry.

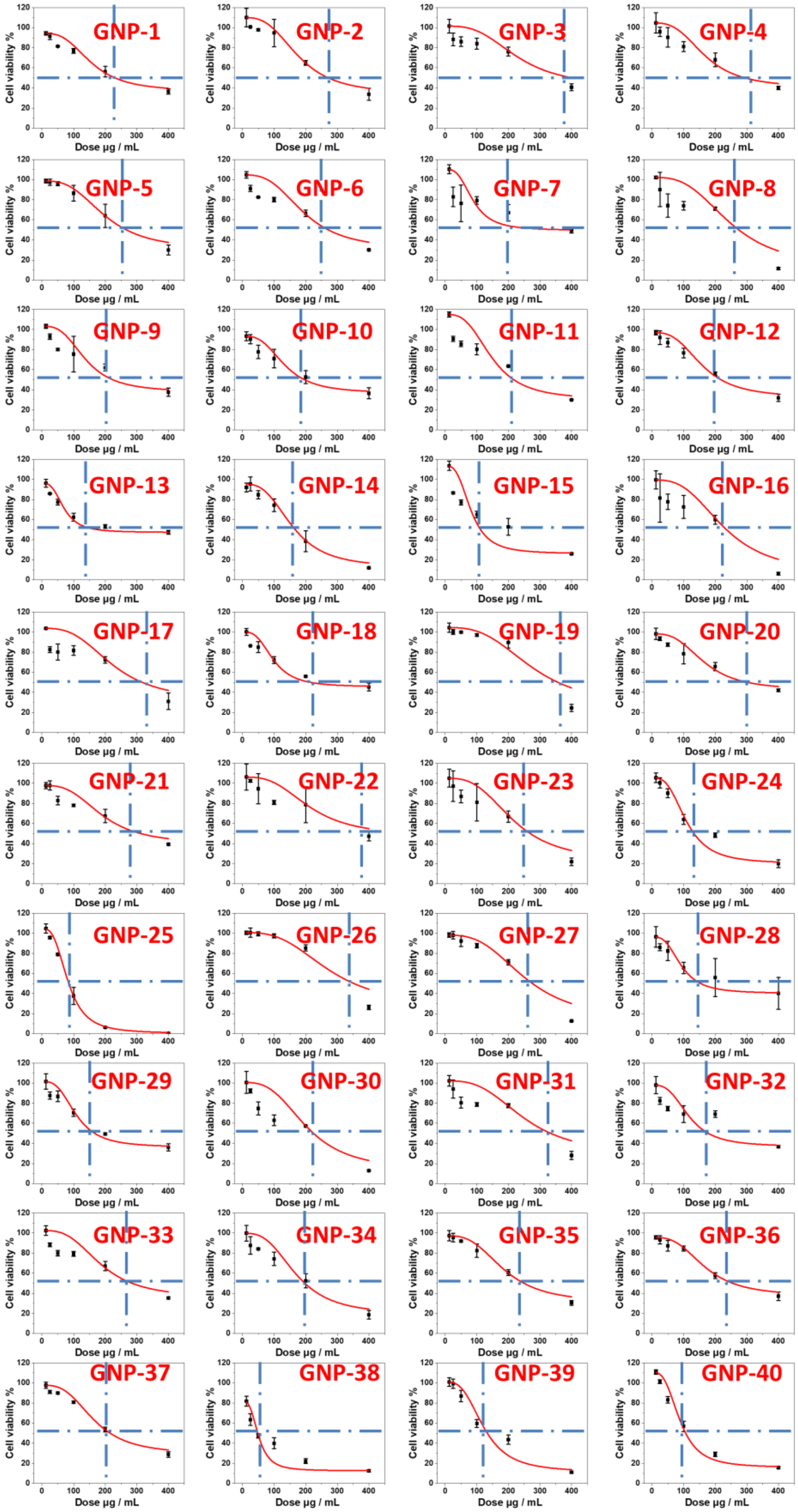
GNP-1
GNP-2
GNP-3
GNP-4
GNP-5
GNP-6
GNP-7
GNP-8
GNP-9
GNP-10
GNP-11
GNP-12
GNP-13
GNP-14
GNP-15
GNP-16
GNP-17
GNP-18
GNP-19
GNP-20
GNP-21
GNP-22
GNP-23
GNP-24
GNP-25
GNP-26
GNP-27
GNP-28
GNP-29
GNP-30
GNP-31
GNP-32
GNP-33
GNP-34
GNP-35
GNP-36
GNP-37
GNP-38
GNP-39
GNP-40
Cell viability %
Dose μg / mL

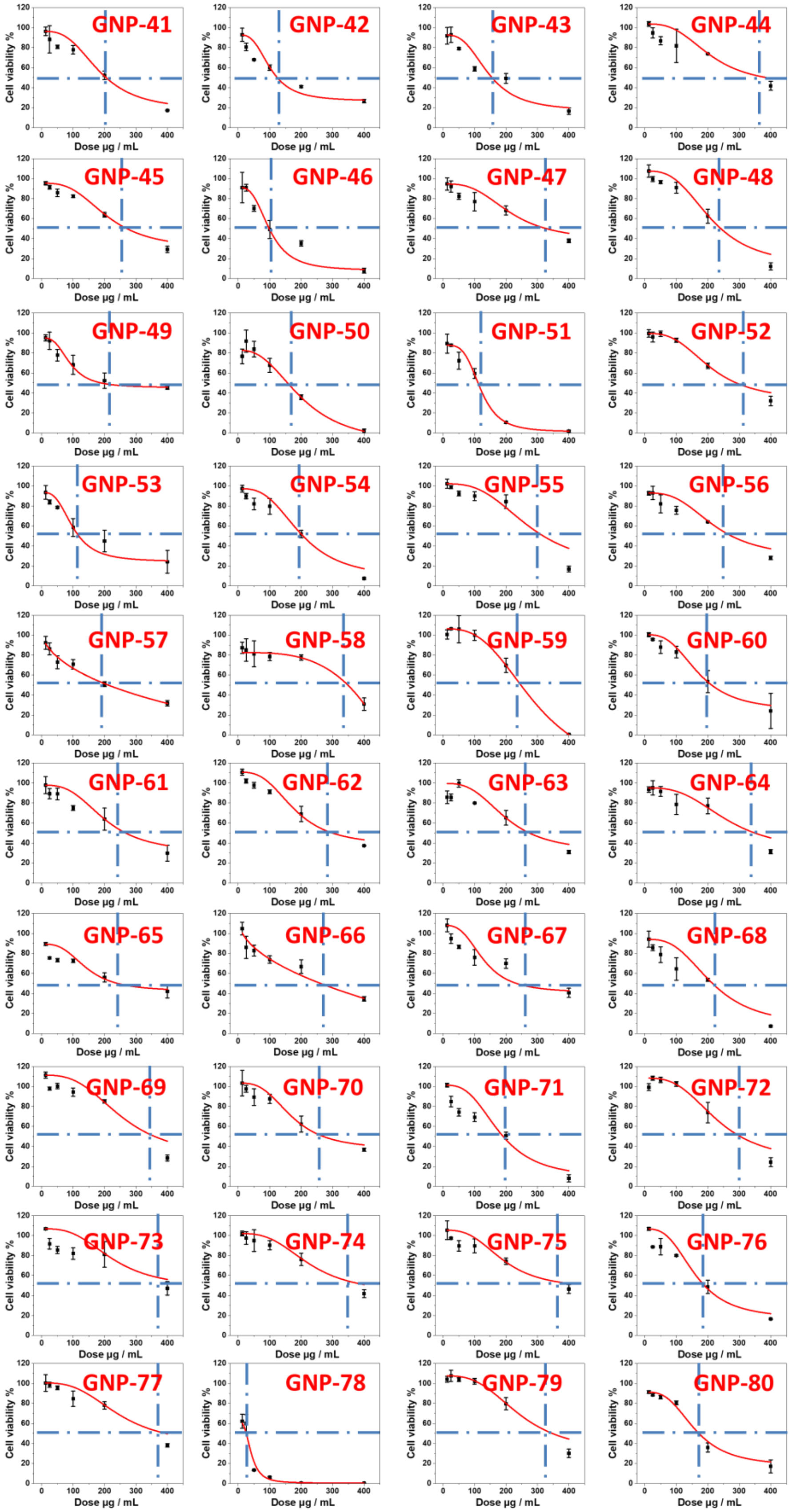

Figure S5. The dose response curves of 80 GNPs in the whole library, blue dotted line:

EC50 labeled.

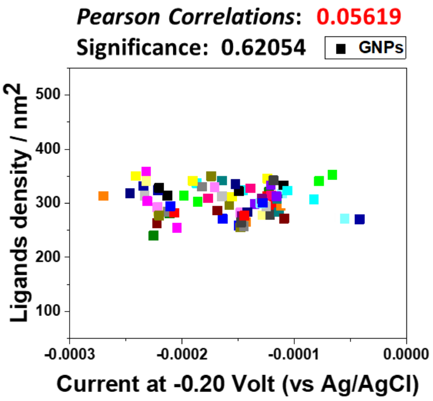

Figure S6. The relationship between the redox activity of 80 GNPs and the number of ligand uploads.

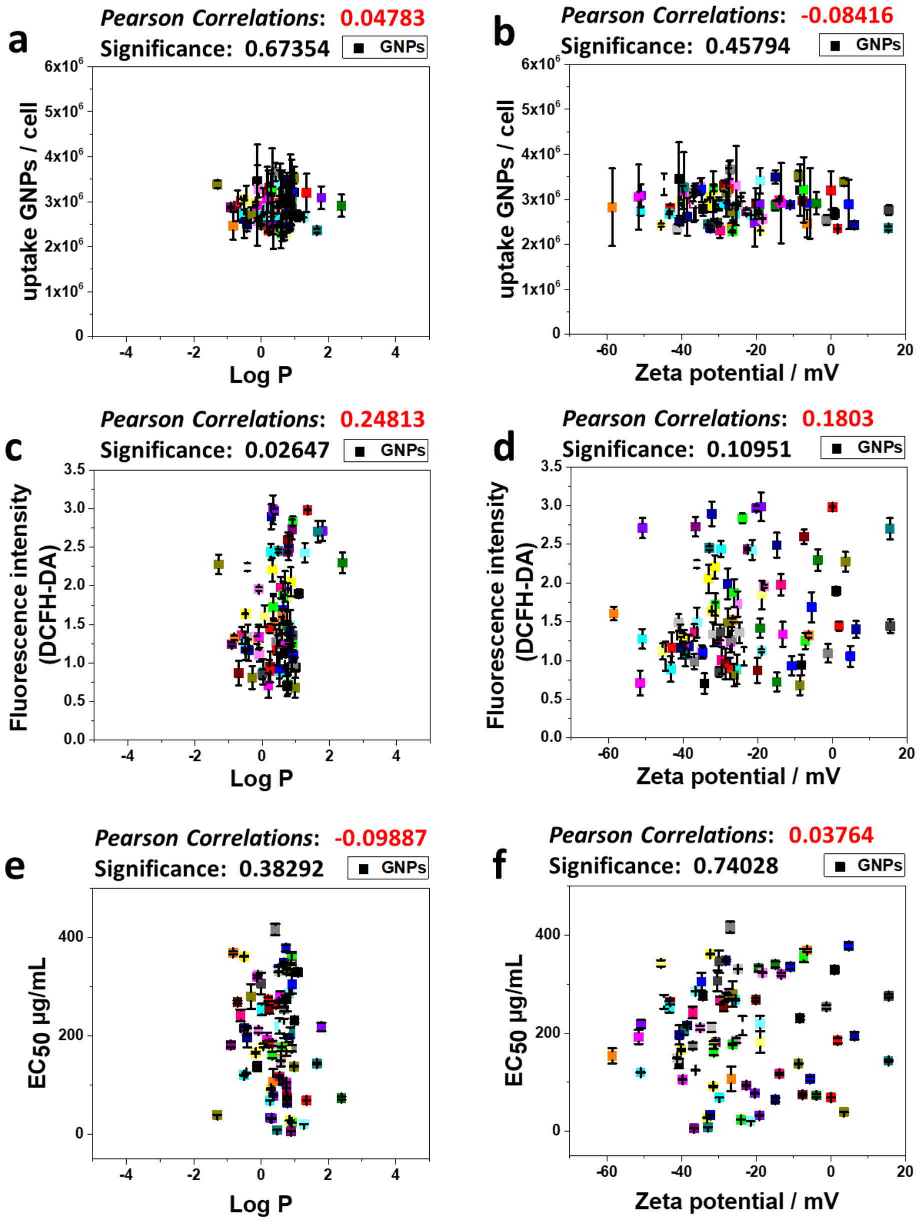

Figure S7. The relationship between the cellular effects of 80 GNPs and hydrophobic properties or surface charge.
